# Supplementary material for: Pouchitis Is Associated with Paneth Cell Dysfunction and Ameliorated by Exogenous Lysosome in a Rat Model Undergoing Ileal Pouch Anal Anastomosis
Source: Microorganisms. 2023 Nov 22;11(12):2832. doi: 10.3390/microorganisms11122832 (PMC10745344; doi:10.3390/microorganisms11122832)
Supplement: Supplementary file 1 [file microorganisms-11-02832-s001.zip › microorganisms-2676882-supplementary.pdf]

Supplementary Table S1: The list of Primers.

Markers	5’-3’
IL-6 forward	GACTTCCAGCCAGTTGCCTTCT
IL-6 reverse	TGGTCTGTTGTGGGTGGTATCC
TNF-α forward	GGGCTCCCTCTCATCAGTTCCA
TNF-α reverse	TGCTCCTCCGCTTGGTGGTT
IFN-γ forward	CCAGGGCCATCAGCAACAACATAA
IFN-γ reverse	CACCGACTCCTTTTCCGCTTC
IL-17 forward	ATCCATGTGCCTGATGCTGTT
IL-17 reverse	GAAGTGGAACGGTTGAGGTAGTC
IL-10 forward	GGGTTGCCAGCCAGTTGCCTTCT
IL-10 reverse	CTTCACCTGCTCCACTGCCTTG
R-Lysozyme forward	CAAGCCATACAATGTGCGAAGAGAG
R-Lysozyme reverse	TGTTGGTTTGAGGGGAAAGCAAG
H-Lysozyme forward	CTTGTCCTCCTTTCTGTTACGG
H-Lysozyme reverse	CCCCTGTAGCCATCCATTCC
R-GAPDH forward	CTGGAGAAACCTGCCAAGTATG
R-GAPDH reverse	GGTGGAAGAATGGGAGTTGCT
H-GAPDH forward	GGAAGCTTGTCATCAATGGAAATC
H-GAPDH reverse	TGATGACCCTTTTGGCTCCC

Figure 1.B

	Fold change of lysozyme	*p* value
TI *vs* NP	1.00±0.00 *vs* 1.35±0.29	0.03
TI *vs* PS	1.00±0.00 *vs* 0.78±0.45	ns
NP *vs* PS	1.35±0.29 *vs* 0.78±0.45	0.04

Figure 2.B

	Control	DSS	*p* value
Pre-pouch	148.58±10.57	162.45±17.32	ns
Pouch	147.94±26.67	74.81±16.06	0.015
p value	ns	0.011	

Figure 2.D

	Control	DSS	*p* value
Normal granule (%)	88.00±5.74	37.20±7.36	0.001

Figure 2F

	Control	DSS	*p* value
Grey scale of lysozyme	0.21±0.09	0.05±0.02	0.05

Figure 2.G

	Control	DSS	*p* value
Fold change of lysozyme	1.00±0.00	0.35±0.30	0.002

Figure 3E

	Fecal score	*p* value
Con *vs* DSS	3.60±0.50 *vs* 2.00±0.70	0.004
DSS *vs* DSS+lyso	3.60±0.50 *vs* 3.20 ± 0.70	0.326
DSS *vs* DSS+lyso	2.00±0.70 *vs* 3.20 ± 0.70	0.005

Figure 3.F

	Histopathological score	*p* value
Con *vs* DSS	7.00 ±0.80 *vs* 11.10±1.00	0.001
Con *vs* DSS+lyso	7.00 ±0.80 *vs* 5.20±0.80	0.03
DSS *vs* DSS+lyso	11.10±1.00 *vs* 5.20±0.80	0.001

Figrue 4.B

	$CD3^+$ cell count	*p* value
Con *vs* DSS	88.30±37.00 *vs* 234.30±25.90	0.005
Con *vs* DSS+lyso	88.30±37.00 *vs* 189±24.30	0.023
DSS *vs* DSS+lyso	234.30±25.90 *vs* 189±24.30	0.130

Figrue 4.C

	$CD45^+$ cell count	*p* value
Con *vs* DSS	46.30±6.40 *vs* 318.30±43.80	0.001
Con *vs* DSS+lyso	46.30±6.40 *vs* 152.60±46.90	0.010
DSS *vs* DSS+lyso	318.30±43.80 *vs* 152.60±46.90	0.011

Figrue 4.E

	Grey scale of Occludin	*p* value
Con *vs* DSS	0.32±0.07 *vs* 0.05±0.02	0.003
Con *vs* DSS+lyso	0.32±0.07 *vs* 0.12±0.04	0.023
DSS *vs* DSS+lyso	0.05±0.02 *vs* 0.12±0.04	0.030

Figrue 4.F

	Grey scale of ZO-1	*p* value
Con *vs* DSS	0.13±0.01 *vs* 0.02±0.02	0.001
Con *vs* DSS+lyso	0.13±0.01 *vs* 0.13±0.04	0.570
DSS *vs* DSS+lyso	0.02±0.02 *vs* 0.13±0.04	0.010

Figrue 4.H

	Fold change of TNF-α	*p* value
Con *vs* DSS	1.00±0.00 *vs* 2.320±0.14	0.013
Con *vs* DSS+lyso	1.00±0.00 *vs* 0.81±0.12	0.530
DSS *vs* DSS+lyso	2.320±0.14 *vs* 0.81±0.12	0.009

Figrue 4.I

	Fold change of IL-6	*p* value
Con *vs* DSS	1.00±0.00 *vs* 1.32±0.14	0.016
Con *vs* DSS+lyso	1.00±0.00 *vs* 0.68±0.32	0.390
DSS *vs* DSS+lyso	1.32±0.14 *vs* 0.68±0.32	0.035

Figrue 4.J

	Fold change of INF-γ	*p* value
Con *vs* DSS	1.00±0.00 *vs* 1.82±0.47	0.039
Con *vs* DSS+lyso	1.00±0.00 *vs* 1.21±0.35	0.520
DSS *vs* DSS+lyso	1.82±0.47 *vs* 1.21±0.35	0.073

Figrue 4.K

	Fold change of IL-17	*p* value
Con *vs* DSS	1.00±0.00 *vs* 3.28±1.28	0.004
Con *vs* DSS+lyso	1.00±0.00 *vs* 1.94±1.04	0.031
DSS *vs* DSS+lyso	3.28±1.28 *vs* 1.94±1.04	0.073

Figrue 4.L

	Fold change of IL-10	*p* value
Con *vs* DSS	1.00±0.00 *vs* 0.67±0.15	0.009
Con *vs* DSS+lyso	1.00±0.00 *vs* 1.10±0.36	0.430
DSS *vs* DSS+lyso	0.67±0.15 *vs* 1.10±0.36	0.042

Supplementary Table S2 Details of the microbial changes in each group

Taxon		CTRL_mean	DSS_mean	LYD_mean	CTRL vs DSS_ *p* value	DSS vs LYD_ *p* value
Phylum	Firmicutes	0.358	0.270	0.353	0.132	0.001
	Proteobacteria	0.065	0.132	0.088	0.003	0.010
	Elusimicrobia	0.000	0.010	0.000	0.011	0.014
Family	Enterobacteriaceae	0.023	0.002	0.005	0.027	0.030
	Clostridiaceae	0.018	0.005	0.005	0.055	0.960
	Lachnospiraceae	0.117	0.086	0.125	0.299	0.035
	Lactobacillaceae	0.061	0.051	0.108	0.708	0.027
Genus	Dorea	0.010	0.002	0.008	0.023	0.016
	Blautia	0.023	0.003	0.012	0.030	0.003
	Roseburia	0.014	0.014	0.033	0.981	0.018
	Lactobacillus	0.061	0.051	0.108	0.726	0.022
	Eubacterium	0.005	0.000	0.001	0.004	0.096

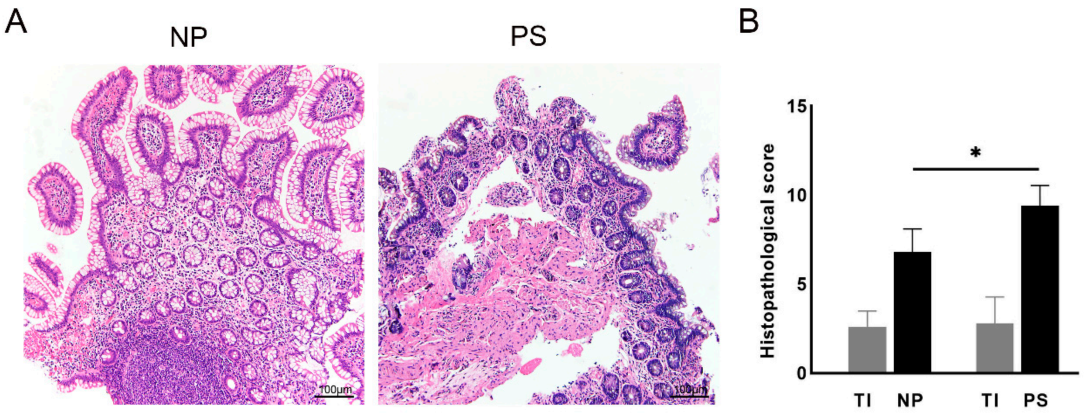

Supplementary Figure S1 A) H & E staining of biopsies tissues (Bar = 100 μ m); B) Histopathological score of each group. HE staining showed mucosal inflammation in pouchitis with a higher histopathological score (6.8±1.3 vs 9.7±1.1, p =0.004) (Figure1.B) Data are shown as the

means±SD with 5 samples on TI, 5 samples on NP and 7 samples on PS. The asterisk indicates a statistically significant difference (*$p < 0.05$.)
